# Zenker’s Diverticulum in Forestier Disease: Chance or Causality?

**DOI:** 10.22038/IJORL.2021.60053.3068

**Published:** 2022-03

**Authors:** Carmelo Saraniti, Giuseppe Greco, Barbara Verro, Enzo Chianetta, Antonio Lo-Casto

**Affiliations:** 1 *Department of Biomedicine, Neuroscience and Advanced Diagnostic, University of Palermo, Palermo, Italy.*

**Keywords:** Diffuse idiopathic skeletal hyperostosis, Dysphagia, Zenker’s diverticulum, Esophageal diverticulum, Forestier disease

## Abstract

**Introduction:**

Zenker's diverticulum is an acquired sac-like outpouching of the mucosa and submucosa layers originating at the pharyngoesophageal junction. The predominant symptom of Zenker’s diverticulum is dysphagia. Videofluoroscopy confirms the diagnosis. Forestier disease is a clinical entity characterized by ossification of anterolateral vertebral ligament and anterior osteophyte formation along the anterolateral spinal column. Its etiopathogenesis remains unknown and common symptoms are dysphagia, dysphonia and airway obstruction. The objective of this study is to identify a pathophysiological correlation between Forestier disease and the onset of Zenker’s diverticulum.

**Materials and Methods:**

A retrospective observational study was conducted. The electronic database of our Radiology Unit was analyzed in order to identify patients with hypopharyngeal diverticulum and osteophytes at the cervical vertebrae level, from January 2010 to January 2021. The search was performed using precise keywords.

**Results:**

The computerized database search outlined 10 imaging exams: 5 videofluorographies and 5 computed tomography scans. In 100% of the cases, dysphagia was the main symptom that led to the diagnostic assessment; 30% of patients, on the other hand, reported dyspnoea. From the data analysis, the male / female ratio is 1: 1 and the average age of the patients is 64.8 (+/- 11.31) years.

**Conclusions:**

We assume that the anatomical abnormalities in Forestier disease may cause an increase of pharyngeal pressure and consequently support the development of the Zenker’s diverticulum. Hence, it is always recommended to investigate the presence of Zenker’s diverticulum in a patient with Forestier disease, especially for the life-threatening complications of Zenker’s diverticulum.

## Introduction

Zenker’s diverticulum (ZD), also known as hypopharyngeal diverticulum, is an acquired prolapse of the mucosal and submucosal layers (false diverticulum) of the posterior hypopharyngeal wall located in a specific area between horizontal fibers of the cricopharyngeal muscle and the oblique fibers of the inferior constrictor muscle; this space is called Killian’s triangle and represents a *locus minoris resistentiae *(1). Ludlow was the first to describe it in 1767and Friedrich Albert von Zenker investigated his pathogenesis about a century later and gave it his name (2,3). 

The annual incidence of ZD is estimated to be 2 in every 100,000 patients and generally affects elderly males (4). The pathogenesis of ZD is unclear even today. One widely accepted theory is an increase of hypopharyngeal pressure during swallowing due to an incomplete relaxation of the cricopharyngeal muscle and subsequent protrusion of mucosa and submucosa dorsally through the above-mentioned weakness area (5,6). 

Dysphagia is the predominant symptom and is described in 80% to 90% of patients. Other symptoms are regurgitation, halitosis, chronic cough and aspiration of food especially during the night with possible episodes of pneumonia. The most severe complications are cancer, hemoptysis or hematemesis, empyema and perforation (7). 

Endoscopic (rigid or flexible) or open surgery are the treatment options for symptomatic cases. In a recent review an endoscopic approach is a faster procedure with shorter hospitalization, earlier diet introduction, and lower rates of complications, but higher rates of symptom recurrence (8). Diffuse Idiopathic Skeletal Hyperostosis (DISH) or Forestier disease is a clinical entity characterized by ossification of anterolateral vertebral ligament and anterior osteophyte formation along the anterolateral spinal column (9). 

Although it is often undiagnosed, DISH affects caucasian race most frequently with a prevalence estimated at 10% to 35% of male patients older than 70 years (10). The etiopathogenesis of DISH remains unknown although risk factors seem to be advanced age, metabolic derangement (hypertension, obesity, diabetes mellitus) and cardiovascular disease (9,11). When the osteophytes originate from the cervical vertebrae, specific symptoms may arise like dysphagia, pharyngeal globus, dysphonia, stridor and airway obstruction (12).

It is known that an increase in intraluminal pressure is at the basis of the origin of ZD. Currently, in literature, DISH is not mentioned among the causes of increased intraluminal hypopharyngeal pressure.

The objective of this research is to identify a pathophysiological correlation between Forestier disease and the onset of Zenker’s diverticulum by means of a retrospective observational study.

## Materials and Methods

*Search methodology*

A retrospective observational study has been carried out. The electronic database of our Radiology Unit was analyzed in order to identify patients with hypopharyngeal diverticulum and osteophytes at the cervical vertebrae level, from January 2010 to January 2021. 

The search was performed using these keywords: *pharyngeal diverticulum,* *esophageal diverticulum *or* Zenker’s diverticulum* and *cervical osteophytes *or* Forestier disease*. Afterwards, an expert radiologist reviewed the selected images (neck-chest computed tomography CT and esophageal videofluorography) to confirm the diagnosis of Zenker's diverticulum and Forestier's syndrome. Inclusion criteria were: (1) both males and females; (2) presence in the report of combinations of keywords; (3) patients undergoing neck-chest CT and/or esophageal videofluorography; (4) confirmation of Zenker's diverticulum and Forestier disease on CT and / or esophageal videofluorography images. Exclusion criteria were: (1) presence of isolated Zenker diverticulum on reports; (2) presence of isolated Zenker diverticulum on CT and/or esophageal videofluorography images; (3) presence of isolated Forestier disease on reports; (4) presence of isolated Forestier disease on CT and/or esophageal videofluorography images. The study was approved by the Ethical Committee (approval number 04/2021).

***Data analysis***

Information on the sex and age of the patients, the symptoms, the types of imaging that have been performed and the location of the osteophytes were collected on an Excel spreadsheet. These data were reported as an integer, a percentage and as an average value (+/- standard deviation).

## Results

**Fig 1:**
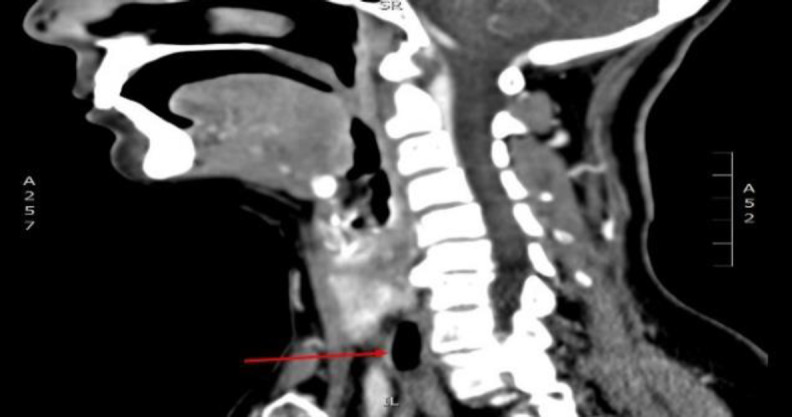
Neck CT (sagittal scan) showing DISH at C5-C6 level and Zenker's diverticulum at C7 level (red arrow)

**Fig 2:**
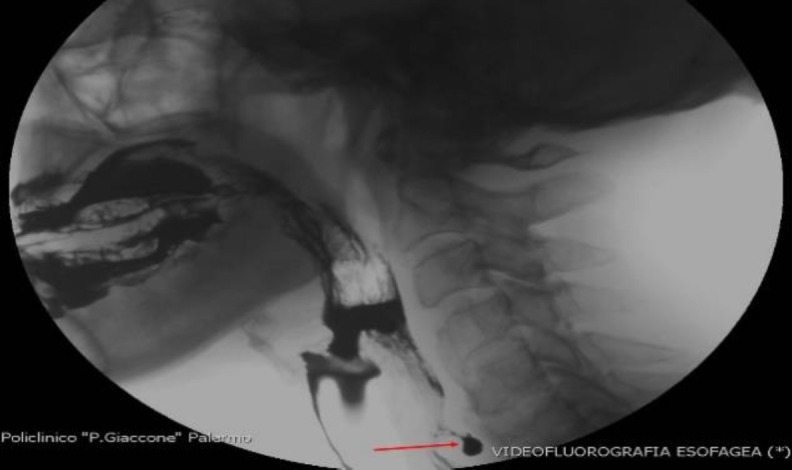
Esophageal videofluorography (lateral scan) showing DISH at C4-C5 level and Zenker's diverticulum at C6 level (red arrow)

The analysis on the computerised database revealed 15 reports. Following the review by an expert radiologist, only 10 imaging tests were selected: 5 videofluorographies and 5 CT scans (Fig. 1-2). 

In 100% of the cases, dysphagia was the main symptom that led to the diagnostic assessment; 30% of patients, on the other hand, reported dyspnoea. From the data analysis, the male / female ratio is 1: 1 and the average age of the patients is 64.8 (+/- 11.31) years. 

The imaging examination showed the presence of DISH more often at the level of C5-C6 (30%).

We have reported below two particular and serious cases of Zenker diverticulum in Forestier disease. The data are represented in Table 1.

**Table 1:** Patients’ characteristics

**Characteristics**	**N° **	** (%)**
Age at diagnosis (years) Mean Range	64.8 (+/- 11.31) 44 - 83	
Gender Female Male	5 5	50 50
Level of osteophytes C4-C7 C4-C5 C5-C6 C6-C7 C3-T1 C6-T1	1 2 3 2 1 1	10 20 30 20 10 10
Symptoms Dysphagia Odynophagia Regurgitation Dyspnoea Cough Weight loss	10 3 2 3 2 3	100 30 20 30 20 30
Total	10	100
		

***Case 1***

**Fig 3:**
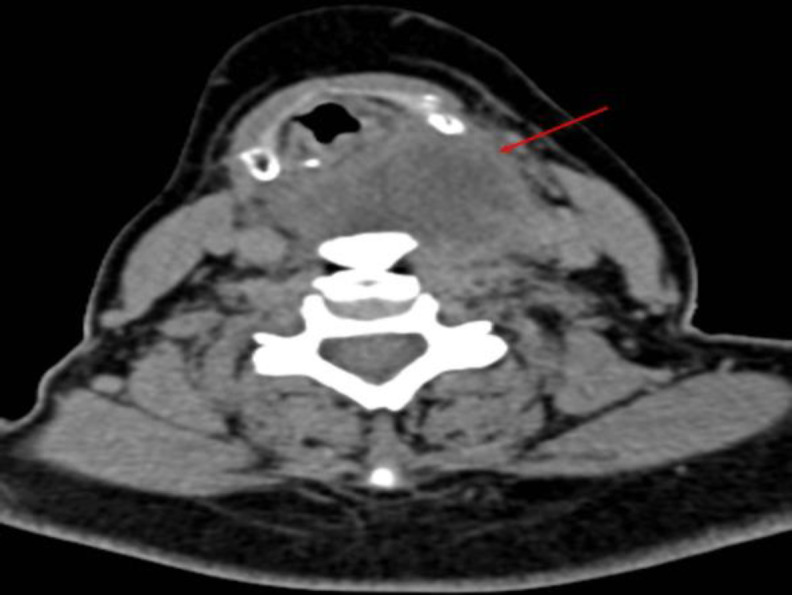
Neck CT (axial scan) showing suppurated diverticulum at the level of C6-C7 (red arrow) and DISH C6-T1 level

A 61-year-old woman had been accepted in the emergency room for dyspnoea and dysphagia, which had worsened in the last 24 hours. The endoscopic examination showed a rotation and lateralization of the laryngeal-tracheal axis to the right with bilateral obstructive arytenoid edema and swelling of the posterior and left lateral wall of the hypopharynx. Neck CT with contrast showed a left retropharyngeal collection (6 cm of cranio-caudal diameter, 3.5 cm antero-posterior, 5 cm transverse) with hyperintense walls and the presence of Forestier syndrome at the level of C6-T1 (Fig. 3). 

Afterwards, the patient underwent a tracheostomy under local anaesthesia for difficult intubation and transoral marsupialization of the diverticular pouch. 2 months later after surgery, the neck CT showed the complete disappearance of the diverticular bag.

***Case 2***

**Fig 4:**
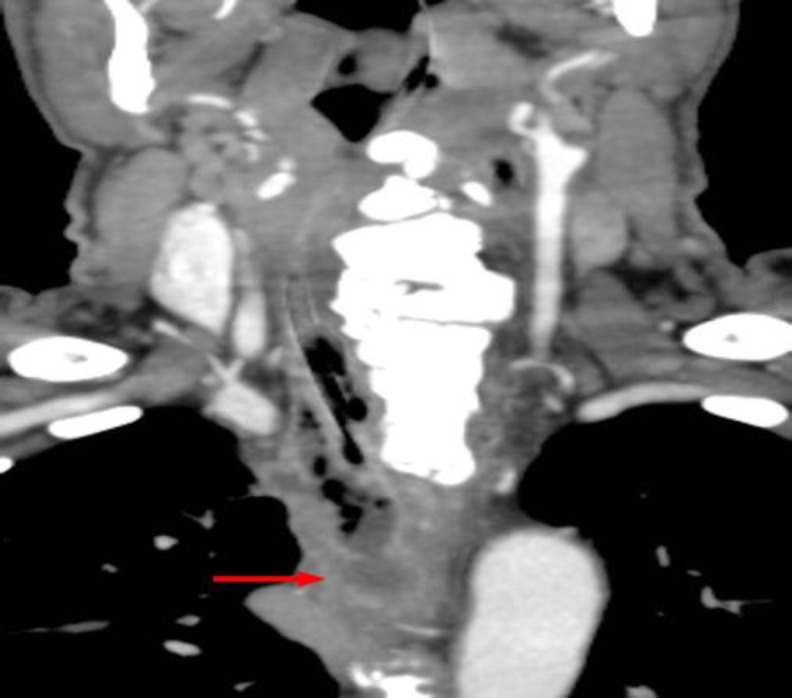
Neck CT scan (coronal scan) showing DISH at the level of C3-T1 and suppurated and fissured diverticulum up to the level of T2 (red arrow)

**Fig 5:**
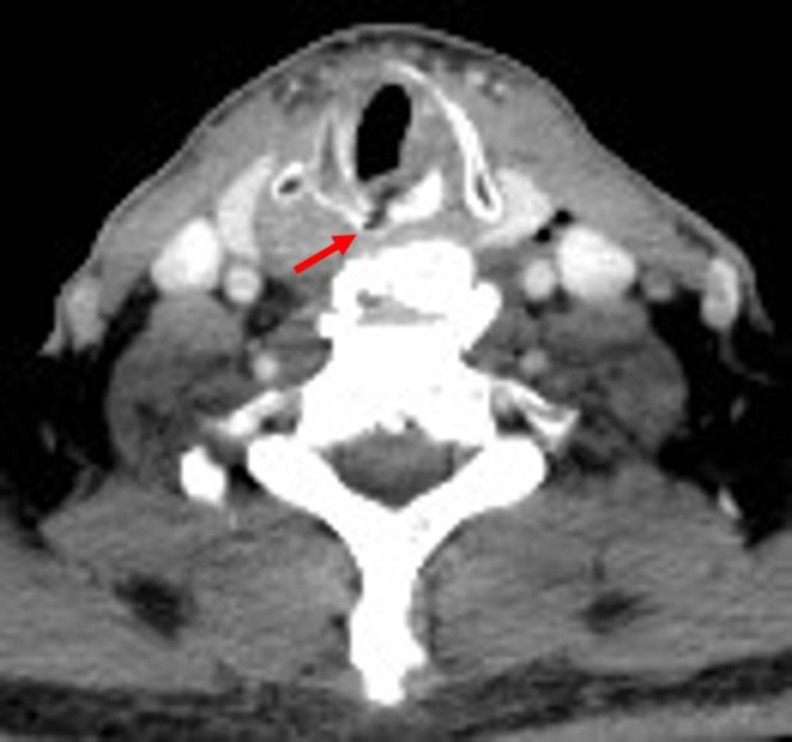
Neck CT (axial scan) showing the fracture of the cricoid plate (red arrow)

A 58-year-old man came to our emergency room for acute dyspnoea and fever for about 24 hours and worsened with dysphagia, already present for several months. The endoscopy revealed diffused laryngeal occlusive edema, swelling of the posterior wall of the hypopharynx and purulent exudate. The patient then underwent an emergency tracheostomy. Subsequently, he performed a neck CT with medium contrast which revealed a suppurated and perforated ZD; anterior paravertebral ossifying hyperostosis between C3 and T1 (Fig. 4) and fracture of the cricoid plate (Fig. 5).

## Discussion

Among the diverticula of the upper gastrointestinal tract, Zenker’s diverticulum is the most common. Characteristic symptom of ZD is dysphagia (for solid and liquid), but in some cases, regurgitation, chronic coughing, hoarseness, halitosis and gastro-esophageal reflux symptoms may occur. ZD usually occurs in hypopharyngeal Killian’s triangle. It is located in the posterior wall of the hypopharyngeal-esophageal junction, between the oblique fibers of the lower constrictor pharyngeal muscle and the transverse fibers of the cricopharyngeal muscle. Its etiopathogenesis is still unclear, but several theories have been proposed over the years. Zenker was the first to propose that the pathophysiological mechanism of the diverticulum has its fulcrum in the increase of endoluminal pressure with progressive exhaustion and herniation of the mucous and submucosal layer of the wall through the muscular tunic of the bowel (3). 

Nonetheless, there is currently no clear interpretation of the causes of this process. In fact, the causes are manifold and different from patient to patient. Furthermore, anatomical predisposition was hypothesized as the causal factor of this condition (13). Some studies of the last decades have also reported pharyngeal diverticulum development as complication after anterior cervical discectomy and fusion (ACDF) (14-18). Therefore, from the analysis of this literature and the assessment of comorbidity characteristics of our patients, we formulated a new etiopathogenetic hypothesis: Forestier's disease (DISH of the cervical spine) could be considered another predisposing factor of Zenker's diverticulum. Diffuse idiopathic skeletal hyperostosis (DISH) is a systemic condition characterized by the ossification of ligaments and entheses in the prevertebral regions of the spine (9). The etiopathogenesis is still partially unknown, despite the fact that it is widely ascertained that a relevant role in pathogenesis is played by presence of endogenous dysmetabolism that predispose to an ossification diathesis (19). 

Indeed, correlations have been found between the onset of disease and diabetes mellitus, obesity, old age, hyperuricemia, hypertension, hyperinsulinemia and elevated insulin-like growth factor (20). 

DISH is generally an incidental finding on imaging. Many classifications were formulated for DISH diagnosis over the years; the more accepted criteria were described in 1976 by Resnick and Niwayama (21) and are as follows: 1) ossification and calcification of the anterolateral paravertebral ligaments along four or more contiguous vertebral bodies; 2) relative preservation of intervertebral disc height; 3) absence of sacroiliac erosion, joint fusion, and apophyseal ankylosis. One of the more affected skeletal segments is cervical spine; patients may complain of neck pain, dysphagia and in rare cases dyspnoea especially when low cervical level (between C4 and C7) is involved (10). In this study we tried to define a potential subset of people who can be more at risk because of sex or age. We found a homogeneous distribution in both sexes, with an onset of symptoms at the age of 64.8 (+/- 11.31). Consistent with the literature, dysphagia symptom was reported by 100% of the patients and rarer but still present was also dyspnoea (20%). There are some limitations on the study. Firstly, a reduced number of observations due to the low incidence of these two syndromes, even more if associated. 

The recruitment of the patients was based on searching for keywords in the reports of CT and videofluorography: therefore, some cases of ZD and / or DISH may have not been recognized and not been included in the reports. In addition, another limitation of this study is that all patients included in the study never underwent hypopharyngeal manometry in order to objectively and instrumentally confirm our hypothesis. In fact, our hypothesis assumes that the presence of osteophytes at the cervical level (Forestier's syndrome) may be a possible cause of the increase of intraluminal pharyngeal pressure which is responsible for the development of Zenker's diverticulum. This correlation is currently absent in the literature.

## Conclusion

The increase of hypopharyngeal pressure is one of the most accredited pathogenetic theories underlying the diverticulum expansion. Our study has assumed and proposed another hypothesis to be the cause of the ZD, never mentioned in literature: the anatomical abnormalities in Forestier disease may cause an increase of pharyngeal pressure and consequently favouring the onset of ZD. Therefore, it is always recommended to investigate the presence of a ZD in a patient with Forestier disease, especially for the life-threatening complications of ZD.

This study may be the input for further studies and researches with a larger population that can confirm our hypothesis.
